# A new approach for incorporating ^15^N isotopic data into linear inverse ecosystem models with Markov Chain Monte Carlo sampling

**DOI:** 10.1371/journal.pone.0199123

**Published:** 2018-06-18

**Authors:** Michael R. Stukel, Moira Décima, Thomas B. Kelly

**Affiliations:** 1 Department of Earth, Ocean, and Atmospheric Science, Florida State University, Tallahassee, FL, United States of America; 2 Center for Ocean-Atmospheric Prediction Studies, Florida State University, Tallahassee, FL, United States of America; 3 National Institute of Water and Atmospheric Research (NIWA), Wellington, New Zealand; Fred Hutchinson Cancer Research Center, UNITED STATES

## Abstract

Oceanographic field programs often use δ^15^N biogeochemical measurements and *in situ* rate measurements to investigate nitrogen cycling and planktonic ecosystem structure. However, integrative modeling approaches capable of synthesizing these distinct measurement types are lacking. We develop a novel approach for incorporating δ^15^N isotopic data into existing Markov Chain Monte Carlo (MCMC) random walk methods for solving linear inverse ecosystem models. We test the ability of this approach to recover food web indices (nitrate uptake, nitrogen fixation, zooplankton trophic level, and secondary production) derived from forward models simulating the planktonic ecosystems of the California Current and Amazon River Plume. We show that the MCMC with δ^15^N approach typically does a better job of recovering ecosystem structure than the standard MCMC or L_2_ minimum norm (L2MN) approaches, and also outperforms an L2MN with δ^15^N approach. Furthermore, we find that the MCMC with δ^15^N approach is robust to the removal of input equations and hence is well suited to typical pelagic ecosystem studies for which the system is usually vastly under-constrained. Our approach is easily extendable for use with δ^13^C isotopic measurements or variable carbon:nitrogen stoichiometry.

## Introduction

Reconstructing ecosystem structure and trophic flows through planktonic ecosystems is crucial for understanding fisheries production, pelagic biogeochemistry, and the response of each of these to a changing climate. However, quantitative investigation of energy transfer between plankton functional groups is hampered by methodological limitations in separating ecological groups and in making rate measurements on specific trophic levels. As a result, oceanographers often rely on mass-balance ecosystem models such as EcoPath [1] or Linear Inverse Ecosystem Models (LIM [2, 3]) to reconstruct trophic structure from sparse measurements. However, the paucity and poor taxonomic resolution of common marine ecological measurements leaves such modeling approaches vastly under-constrained (e.g. [4]). Stable isotope signatures (δ^15^N and δ^13^C) offer additional information on diet and trophic position but are limited by difficulties in physically separating plankton functional groups with overlapping size [5–7]. Additionally, in contrast to terrestrial or estuarine systems where bulk stable isotopes of different primary producers are very different (e.g. depending on C3 or C4 plant metabolism), such differing bulk values have not been observed within plankton size classes [8], although arguably this could also be due to the size overlap of organisms with different isotope values [9]. Approaches capable of combining isotopic data and mass-balance approaches are thus clearly needed [10].

Although the frequency of *in situ* pelagic ^15^N isotopic measurements is increasing, these data are seldom incorporated into inverse ecosystem models for two related reasons. First, although δ^15^N measurements are tractable for several of the inputs and outputs to the planktonic ecosystem (e.g. metazoan zooplankton and larger organisms, sediment trap-based sinking material, NO_3_^-^), few measurements are made of intermediate compartments in the ecosystem, such as different phytoplankton or protozoan taxa, due to the methodological difficulty of these measurements [9], although particulate organic matter (POM) which includes a combination of the mentioned compartments in addition to detritus and bacteria, is often measured as well. This paucity of information on lower trophic levels would necessitate the use of a variable ^15^N isotopic ratio for these taxa, which makes it impossible to linearize the equations. To date, studies incorporating variable isotopic ratios into LIM have included, at most, three compartments with unknown ^15^N [11, 12]. Instead, δ^15^N measurement results are typically used in stable isotope mixing models that often overly simplify the linkages in a planktonic ecosystem [13, 14]. Such models can, for instance, determine the relative role of nitrate uptake and nitrogen fixation in supporting pelagic new production if δ^15^N-NO_3_^-^ and δ^15^N-exported material are measured [15–17]. However, this approach relies on the assumption that sediment trap caught material is representative of the isotopic signature of all material exported from the system, and thus neglects export mediated by larger organisms that are often enriched in ^15^N. Similarly, attempts to estimate fish trophic levels often use simple linear mixed model approaches that assume that specific baseline consumers (e.g. crustacean zooplankton or filter-feeding benthic organisms) are strictly herbivorous (i.e. at a trophic level of two) [18–21] despite the facts that there can be multiple trophic steps within protists and the baseline consumers often feed efficiently on microzooplankton. Powerful new stable isotope models based on Bayesian statistical methods provide an additional approach for incorporating stable isotope measurements with prior knowledge of organism diet and isotopic fractionation [22–24]. However, these approaches are often developed for analyzing only a single trophic step and assume that the stable isotope signature of all dietary sources can be measured (but see [25] for one notable exception). Pacella et al. [26] combined Bayesian and LIM methods by using stable isotope analysis in R (SIAR) to incorporate dual isotopic signatures (δ^13^C and δ^15^N) and constrain the dietary compositions of organisms from a seagrass ecosystem. These dietary compositions then provided powerful additional constraints for the LIM model. However, this approach can only be used for taxa for which the isotopic signatures of all dietary sources are known and hence is not well suited to pelagic ecosystems. An approach capable of linking a trophic and biogeochemical perspective (and assimilating measurements from both disciplines) would clearly be useful.

In this manuscript, we develop a framework for simultaneously incorporating δ^15^N measurements and ecological rate measurements into a LIM while using Markov Chain Monte Carlo (MCMC) sampling to thoroughly sample the under-determined solution space. A scheme previously developed by van Oevelen et al. [12] for incorporating δ^15^N data into LIMs relied on the L_2_ minimum norm (L2MN) approach. However, inherent biases in this L2MN approach [27], suggest that methods reliant on it may be less accurate than approaches using the more recently developed MCMC LIM methodology [28, 29]. Indeed, the L2MN approach has since been shown to be a less robust predictor of ecosystem flows than the MCMC approach [28, 30]. Studies incorporating nitrogen or carbon isotopic information from benthic ecosystems into the MCMC approach have either assumed that the isotopic signature of all compartments within an ecosystem are known [31] or used enriched stable isotope tracer additions to trace mass flow within the ecosystem [32, 33]. Since neither of these approaches is feasible for pelagic systems in which methodological considerations make it impossible to determine the isotopic signatures of many ecosystem compartments, we develop a new approach for incorporating isotopic information into the existing MCMC LIM framework. We follow the approach of Vézina and Pahlow [34] and use forward ecosystem models to generate simulated ecosystem flows; then use these simulated ecosystems to test the efficacy with which our new approach (MCMC+^15^N), the Van Oevelen approach (L2MN+^15^N), and the traditional MCMC and L2MN approaches recover key ecosystem parameters when supplied only with the types of data that are typically collected at sea.

## Methods

We use two different forward models (NEMURO and DIAZO) and two separate sets of boundary conditions for each to create four different sets of fully constrained ecosystem flows. Because LIM models are designed to investigate the static, mass-balanced ecosystem structure, we run each model to steady-state in a simple one-box ecosystem configuration. We then determine input measurements—including ecosystem rate measurements (phytoplankton net primary production, nitrate uptake, mesozooplankton grazing, and sinking particle flux) and δ^15^N values of allochthonous nitrate sources, DOM, mesozooplankton, and sinking particles–that are representative of the types of measurements that can be made in the field. We use these input measurements to constrain LIMs solved using four different approaches: standard L2MN and MCMC approaches and L2MN and MCMC approaches that incorporate δ^15^N data. We then evaluate the ability of these LIM approaches to recover key ecosystem parameters related to nitrogen biogeochemistry (the relative proportion of phytoplankton production supported by new nitrate or nitrogen fixation) and plankton trophic dynamics (mesozooplankton mean trophic level and secondary production). We also conduct additional tests in which fewer ecosystem rate measurements are used as inputs to the inverse model. The following sections explain the forward models used and the four LIM approaches tested.

### Forward models

To generate test datasets with known ecosystem flows (Table 1) we used two published nitrogen-based ecosystem models (NEMURO and DIAZO) with distinctly different model structure and behavior. NEMURO was developed to investigate zooplankton secondary production in the North Pacific for inclusion into ecosystem-based fisheries models [35, 36]. It contains three nutrient pools (nitrate, ammonium, and silicic acid) and two phytoplankton taxa (diatoms and small phytoplankton). It also has a relatively complex zooplankton community including protozoans, small herbivorous mesozooplankton, and large “predatory” mesozooplankton (that are actually omnivorous in the model). We configured the NEMURO model to run in a simple system designed to approximate the ecological dynamics of the euphotic layer in the southern California Current Ecosystem (CCE). Two versions of NEMURO were run, representing the coastal upwelling and offshore oligotrophic regions of the CCE. In the coastal configuration a 20-m deep euphotic zone was parameterized with upwelling rates of 1 m d^-1^, while in the offshore region a 100-m euphotic zone was parameterized with 0.1 m d^-1^ upwelling. We parameterized NEMURO using the phytoplankton values determined by Li et al. [37] from *in situ* rate measurement experiments conducted on CCE Long-Term Ecological Research cruises.

**Table 1:** Comparison of the structures of the two forward (dynamical) models (NEMURO and DIAZO) and the inverse model (LIM).

	Nutrients	Phytoplankton	Zooplankton	Bacteria	Diazotrophy	Configurations
**NEMURO**	NO_3_ NH_4_ Si	Small^S,M^ Large^M,P^	**S**mall (Protists) **M**esozooplankton **P**red. Mesozoo.	Implicit	No	Coastal Offshore
**DIAZO**	Nitrogen Phosphorus Silica	Trichodesmium^P^ Unicellular Diazotrophs^P^ Cyanobacteria^P^ Diatoms^M,P^ DDA^M,P^	**P**rotists **M**esozooplankton	Implicit	Yes	Coastal Mesohaline
**LIM**	NO_3_ NH_4_	Small Phytoplankton^N,μ^ Large Phytoplankton^μ,M^	**N**anoflagellates (μ)Microzooplankton **M**esozooplankton	Explicit	Yes	L2MN L2MN+^15^N MCMC MCMC+^15^N

Under phytoplankton categories, the superscripts denote which zooplankton groups consume the phytoplankton group. The 4 LIM configurations (L2MN, L2MN+^15^N, MCMC, MCMC+^15^N) are each run 4 times to simulate the 4 forward model configurations (NEMURO-Coastal, NEMURO-Offshore, DIAZO-Coastal, DIAZO-Offshore).

The DIAZO model was developed to investigate diazotroph growth and mortality terms in the Amazon River Plume [38]. As a result it has a diverse phytoplankton community including cyanobacteria, unicellular microbial diazotrophs, diatoms, diatom-diazotroph assemblages, and *Trichodesmium*. DIAZO has three nutrient pools (dissolved inorganic nitrogen, dissolved inorganic phosphorus, and silicic acid) and two zooplankton compartments (protozoans and mesozooplankton). Unlike NEMURO, DIAZO also allows protozoans to feed on themselves, thus simulating the longer protozoan food webs found in oligotrophic regions. We configured DIAZO to run in two 1-D systems: a low salinity coastal plume region (with high nutrient concentrations derived from the Amazon River outflow) and a mesohaline region with depleted NO_3_^-^ but high Si and PO_4_^+^ that is believed to be an ideal habitat for diazotrophs [39, 40].

In the DIAZO and NEMURO models, mesozooplankton mortality equations simulate predation by un-modeled higher trophic levels and other mortality terms and return the consumed mesozooplankton nitrogen to the detritus, dissolved organic nitrogen (DON), and dissolved nutrient pools. Since export mediated by sinking mesozooplankton carcasses or un-modeled higher trophic levels (including active transport and fecal pellets produced by fish and squid) is not typically captured in sediment traps, we modified both models such that mesozooplankton biomass consumed by this higher trophic level consumption was simply removed from the model (i.e. mesozooplankton secondary production is a sink term in the planktonic ecosystem).

Since our goal was to investigate the utility of δ^15^N isotopic data for inverse modeling reconstructions of pelagic food webs, we added a nitrogen isotopic model to NEMURO and DIAZO. The isotopic model was based on Yoshikawa et al. [41] and adds extra state variables representing the concentration of ^15^N in each nitrogen-containing model compartment (all living compartments, nutrients, DON, and detritus). Following Yoshikawa et al. [41], phytoplankton take up NO_3_^-^ with a ^15^N fractionation factor (ε_NO3_) of -5‰ and NH_4_^+^ (and dissolved inorganic nitrogen (DIN) in the DIAZO model) with ε_NH4_ = -10‰. Zooplankton excretion and egestion are accompanied by fractionation factors of ε_exc_ = -5‰ and ε_eg_ = -2‰, respectively, while remineralization of detritus or DON to NH_4_^+^ has ε_rem_ = -1‰. We assume that nitrogen fixation (not included in [41]) introduces nitrogen to the ecosystem with a ^15^N isotopic ratio equal to that of atmospheric nitrogen.

The DIAZO and NEMURO models with ^15^N were both run to steady state using a 15-minute time step. Our goal with these simple 1-D models was not to faithfully simulate the full dynamics of the ecosystems modeled (although the steady state solutions agree reasonably with in situ measurements of phytoplankton size structure and growth rates and zooplankton grazing structure measured in the California Current Ecosystem [42–44] or Amazon River Plume [39, 45, 46]). Rather, we wanted to produce simple steady-state inputs representing four distinct ecosystem states (CCE coastal and offshore; Amazon Plume coastal and mesohaline) using models that differed substantially from each other and from the underlying ecosystem structure that we use for the inverse model. This replicates the difficulty encountered when attempting to model an *in situ* ecosystem for which the true structure of the system is unknown and hence the inverse model structure cannot be assumed to perfectly match the *in situ* plankton functional groups.

From these 4 forward simulations we determine steady-state rate values (net primary production, NO_3_^-^ uptake, mesozooplankton grazing, and sediment trap-derived export), standing stocks (cyanobacteria biomass, diatom biomass, mesozooplankton biomass), and δ^15^N values (of exogenous NO_3_^-^ entering the ecosystem, mesozooplankton, DOM, and sinking detritus) that are representative of measurements that can be readily made in the field. We use these values as input measurements for the LIM approaches (see below). We then assess each LIM approaches’ ability to recover 15 withheld forward model results for each forward model configuration: N_2_ fixation, total phytoplankton nitrogen uptake, cyanobacteria net primary production (NPP), diatom NPP, protozoan grazing rate (on phytoplankton), mesozooplankton carnivory (on other mesozooplankton), protozoan carnivory (on other protists), mesozooplankton secondary production, protozoan gross growth efficiency (GGE), mesozooplankton GGE, protozoan trophic level (TL), mesozooplankton TL, herbivorous food chain (the percentage of total NPP that mesozooplankton consume through direct grazing of phytoplankton), multivorous food chain (proportion of NPP that is consumed in a food chain including multiple zooplankton groups before contributing to higher trophic levels), microbial loop food web (ratio of bacterial remineralization of DOM to total NPP). Note that bacterial remineralization is implicit in both forward models and explicit in the LIM models.

### Linear Inverse Model (LIM) ecosystem structure

**Fig 1:**
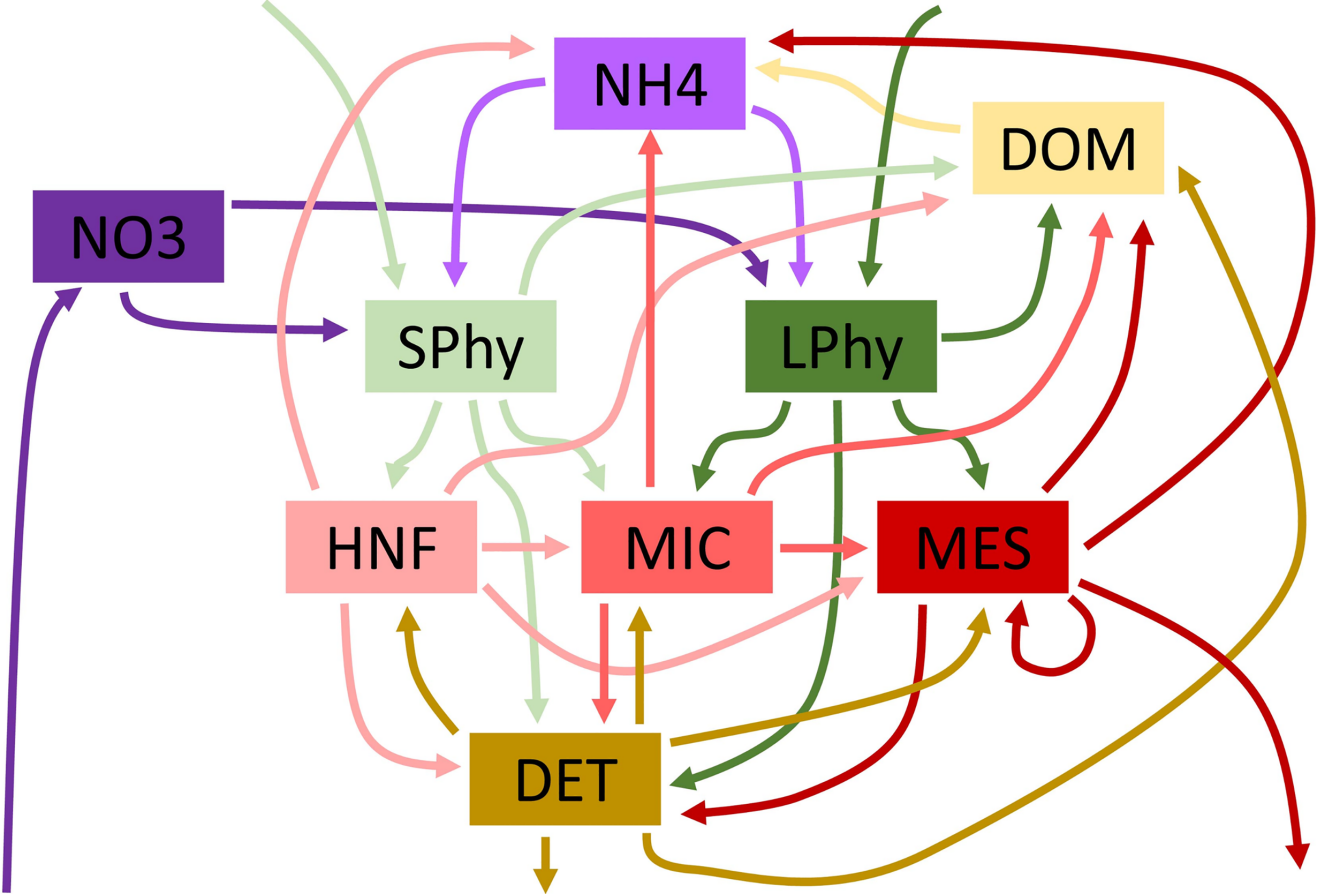
Schematic diagram showing LIM ecosystem flows. Compartments are nitrate (NO3), ammonium (NH4), small phytoplankton (SPhy), large phytoplankton (LPhy), heterotrophic nanoflagellates (HNF), microzooplankton (MIC), mesozooplankton (MES), detritus (DET), and dissolved organic matter (DOM).

**Fig 2:**
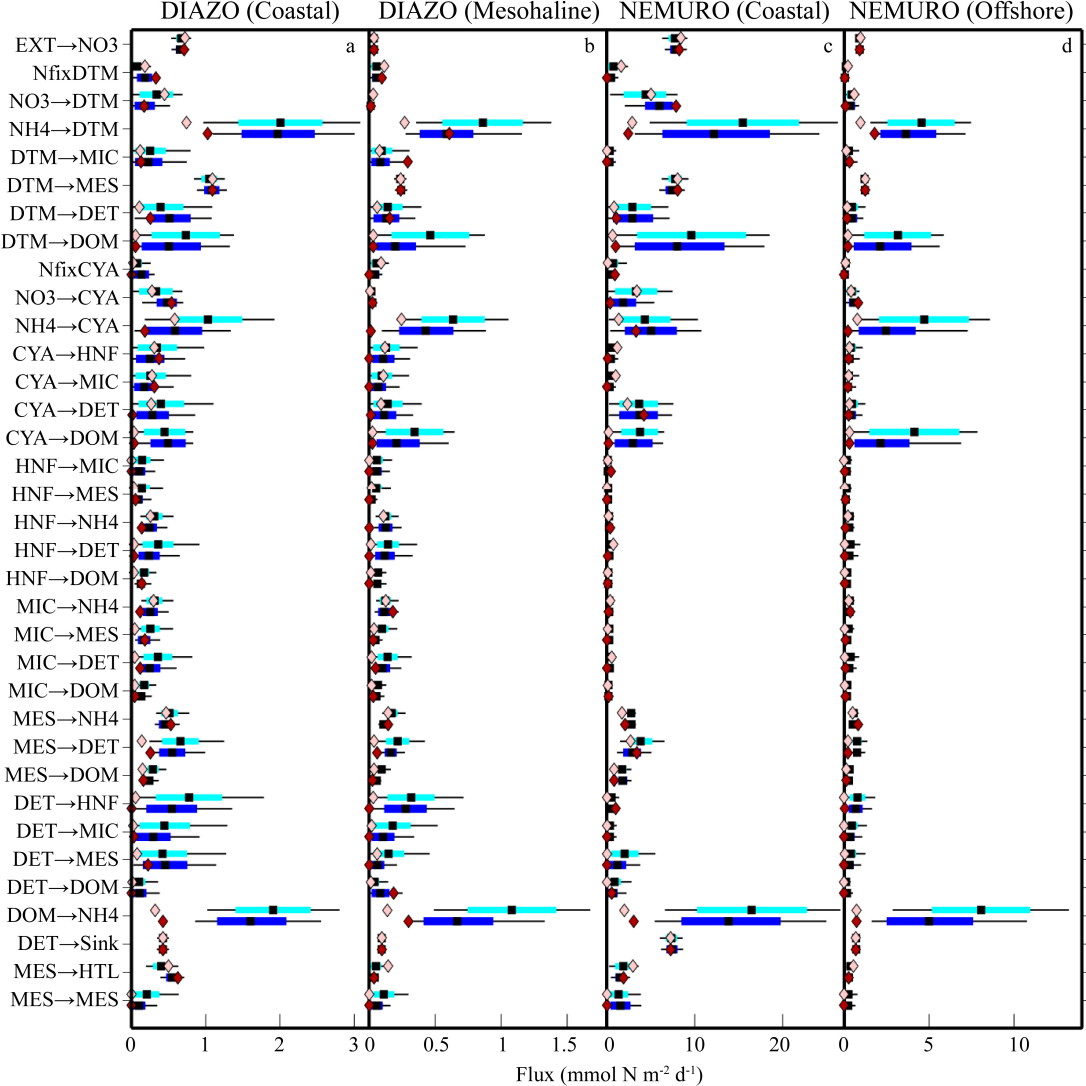
LIM solutions for DIAZO (Coastal) model (a), DIAZO (Mesohaline) model (b), NEMURO (Coastal) model (c), and NEMURO (Offshore) model (d). Units are mmol N m^-2^ d^-1^. Light and dark box plots show 95% confidence intervals, quartiles, and mean for MCMC and MCMC+^15^N, respectively. Light and dark diamonds show values determined by L2MN and L2MN+^15^N, respectively.

We use a simple LIM ecosystem structure that is characteristic of many LIM models of the planktonic ecosystem and borrows elements from Jackson & Eldridge [47] and Richardson et al. [48]. Our LIM includes 5 living compartments: small phytoplankton (SPHY), large phytoplankton (LPHY), heterotrophic nanoflagellates (HNF), microzooplankton (MIC), and mesozooplankton (MES). It also includes 4 non-living compartments: ammonium (NH4), nitrate (NO3), detritus (DET), and dissolved organic matter (DOM). All model flows are measured in nitrogen currency (mmol N m^-2^ d^-1^). Phytoplankton production is supported by uptake of NO3 and NH4, and nitrogen fixation. Heterotrophs graze on phytoplankton (MES are assumed to be incapable of grazing on SPHY) as well as on other heterotrophs smaller than themselves. Mesozooplankton are also assumed to be capable of carnivory on themselves (though other grazers do not feed on themselves). All living taxa lose nitrogen to DOM (excretion) and to DET (defecation by grazers, mortality by phytoplankton). DET is remineralized to DOM and DOM is remineralized to NH4. Inputs of nitrogen to the ecosystem (upwelled or advected NO3 and nitrogen fixation) are balanced by sinking DET and loss of MES to higher trophic levels (HTL). There are a total of 35 ecosystem flows (Figs 1 and 2).

In the LIM we solve a series of systems of equations including:

Ex=f (1)

which quantifies mass balance constraints on each model compartment (9 equations).

Ax≈b (2)

which quantifies measurement constraints with associated uncertainty (primary production, nitrate uptake, mesozooplankton grazing, and sediment trap flux). We assume that measurement uncertainty in these parameters is ±10%. For the MCMC+^15^N and L2MN+^15^N models we also include ^15^N mass balance constraints. These constraints are included in the approximate equations because, while mass balance must hold, we assume nitrogen fractionation factors included in these equations are not exactly known. We assume that uncertainty in these mass balance equations is equal to 10% of the sum of the mass flow through the compartment multiplied by the expected isotopic fractionation between compartments.

Gx≥h (3)

which quantifies *a priori* assumed greater than / less than constraints on organisms within the ecosystem (e.g. gross growth efficiency of grazers varies between 10 and 40%). A full list of greater than / less than equations can be found in the S1 Supplementary Text.

### L2MN and MCMC model approaches

With 35 unknown flows and only 9 exact equalities and 4 approximate equalities (and an additional 10 approximate equalities for the LIM solutions including ^15^N), the model is substantially under-constrained. Hence there are an infinite number of solutions that can satisfy Eqs 1, 2, and 3 (unless some of the equations and inequalities are inconsistent). To choose amongst this infinite number of solutions to the inverse problem, the study that originally brought LIM methodology to aquatic ecology used an L2MN approach [3, 49]. This approach requires that the solution must satisfy Eq 1 and In Eq 3. The L2MN approach then chooses the solution that minimizes Eq 2 and if there are multiple solutions that have zero residual error in Eq 2, the L2MN approach chooses the solution that minimizes the sum of squared flows through the ecosystem. To solve the inverse problem we used the function lsei contained in the R Package limSolve [50].

While the L2MN approach has been widely used, there is no *a priori* reason to assume that the solution that minimizes nitrogen (or carbon) flow through the ecosystem is the most appropriate solution. As an alternate approach, Kones et al. [28, 29] developed a Markov Chain Monte Carlo (MCMC) random walk method that uses a Metropolis algorithm to sample throughout the viable solution space. The MCMC approach initiates with a solution (x_0_) that is known to solve Eqs 1 and 3. A new proposed sample (x_1_) is then drawn from a random jump through the region constrained by Eqs 1 and 3. The residual error of x_1_ with respect to Eq 2 is then compared to the residual error of x_0_ to determine whether the new solution should be accepted. If x_1_ is accepted the process is iterated to determine another solution. If not, the process is repeated from x_0_. In this way a constrained random walk is performed through the solution space. This iteration procedure produces a target distribution of solutions satisfying Eqs 1–3. Summary statistics (mean, standard deviation, confidence intervals) for each ecosystem flow can then be calculated. For more details, we refer readers to Van den Meersche et al. [51] and van Oevelen et al. [2]. The MCMC mean solution has been shown to more accurately estimate in situ ecosystem measurements that are withheld as inputs to the model [30, 52]. It also avoids the undesirable tendency that the L2MN approach has for choosing solutions that are extreme values of the possible solution space (i.e. the L2MN approach often selects solution sets that rest on one of the hyperplanes formed by Gx≥h). We implemented the MCMC approach using the R function xsample [51].

### L2MN+^15^N model approach

Incorporating ^15^N into a LIM is essentially equivalent to incorporating any other form of second currency into the model (e.g. using carbon and nitrogen to constrain the model). Such models are a trivial extension of previous LIM approaches if the stoichiometry (C:N or δ^15^N) of all model compartments is known or assumed. However, in most pelagic ecosystem studies the δ^15^N (or C:N) values of many components of the ecosystem cannot be measured. In a study focused on benthic ecosystems, van Oevelen et al. [12] developed an approach for incorporating δ^13^C measurements into a LIM model for which the δ^13^C of three components of the ecosystem were unknown. Since trophic fractionation of δ^13^C is minimal, they incorporated linear mixing equations into the equality constraints with the following form:

$$\delta^{13}C_{j}=\frac{\sum_{i}^{}\left(\delta^{13}C_{j}\times {flow}_{i\to j}\right)}{\sum_{i}^{}{flow}_{i\to j}}$$ (4)

They then conducted a grid search (±0.1 δ^13^C) calculating the L2MN solution for every reasonable combination of δ^13^C values for the 3 compartments with unknown δ^13^C. The chosen solution was the solution that minimized the residual error and (if multiple solutions had zero residual error) the sum of squared ecosystem flows.

It was necessary to adapt this approach to work with δ^15^N, because trophic fractionation significantly affects the δ^15^N values of a compartment, as they are determined not only by their sources of nitrogen but also by the degree of fractionation of the loss terms from that compartment. Thus we constructed a system of δ^15^N mass balance equations for the flow of ^15^N into and out of each compartment, e.g. for NO3:

$$R_{upno3}\times EXT\to NO3-(R_{no3}\times \alpha_{no3})\times NO3\to CYA-(R_{no3}\times \alpha_{no3})\times NO3\to DTM=0$$ (5)

where R_upno3_ and R_NO3_ are the ratio of ^15^N to total N for allochthonous nitrate entering the ecosystem and euphotic zone nitrate, respectively, α_no3_ is the isotopic fractionation coefficient for nitrate uptake and is equal to exp(ε_no3_), EXT→NO3 is the ecosystem flow corresponding to allochthonous nitrate entering the ecosystem, and NO3→CYA and NO3→DTM are the ecosystem flows corresponding to nitrate uptake by CYA and DTM. The full set of ^15^N mass balance equations can be found in Appendix 1. These equations were incorporated into the approximate equations (Eq 2), because while mass balance must hold, we assume that fractionation terms are uncertain.

We included the δ^15^N values of allochthonous nitrate entering the ecosystem, euphotic zone DOM, zooplankton, and sinking detritus as measured inputs given to the LIM, because these measurements can be readily made in the field (e.g. [53–56]). This left 6 model compartments (NO3, NH4, CYA, DTM, HNF, and MIC) for which δ^15^N was unknown. We conducted a grid-search through this 6-dimensional grid space testing all realistic parameter ranges (with 0.25 ‰ step size), solving the L2MN for each δ^15^N parameter set. The solution set with the lowest residual norm (*σ*^-2^(*Ax-b*)^T^(*Ax-b*)) was selected as the L2MN+^15^N solution. We note, however, that a slightly better solution might be found with greater discretization of the tested δ^15^N values although computational power limited the step size we could use (decreasing the step size from 0.25 to 0.1 would have required computing the L2MN ~20 billion times rather than ~100 million times). Use of the L2MN+^15^N approach with models containing additional compartments will likely require the use of a gradient-based variational approach rather than a full grid search.

### MCMC+^15^N model approach

The MCMC approach has many desirable qualities, not least of which is the fact that it allows computation of model uncertainty resulting from both uncertainty in the inputs to the model and the inherent under-determinacy of the system [28]. The MCMC is also a more efficient sampler of the parameter space than a full grid search and we took advantage of this by including varying δ^15^N values in the MCMC search algorithm. These unknown δ^15^N values are stored in a new vector (∂_U_), while the known (measured) δ^15^N values are stored in a vector (∂_K_). The δ^15^N values are incorporated into the approximate equations (Eq 2) using the same mass balance constraints as for the L2MN+^15^N (Appendix 1). The matrix A is thus now a function of ∂_U_ and ∂_K_. During the MCMC algorithm when a new proposed sample (x_1_) is determined with a random jump from the previous solution vector (x_0_), we also propose a new set of δ^15^N values (∂_U,1_) by performing a random jump from the prior set of δ^15^N values (∂_U,0_). We then call a function that recalculates the matrix A_1_ as a function of ∂_U,1_ and ∂_K_. We then proceed (as for the standard MCMC approach) to decide whether to accept or reject x_1_ and ∂_U,1_ based on the ratio of p(x_1_,A_1_)/p(x_0_,A_0_). If the values are accepted, both x_1_ and ∂_U,1_ are appended to the overall solution. If not, they are rejected and the process is repeated from x_0_ and ∂_U,0_. This approach thus generates a series of solutions that satisfy the equality constraints (Eq 1) and inequality constraints (Eq 3), while approximately satisfying the input measurements and δ^15^N mass balance equalities codified in Eq 2 and Appendix 1. Model code was written in the open source language R (3.3.2) to take advantage of existing algorithms in the limSolve package [57] and can be downloaded from GitHub at: https://github.com/stukel-lab/N15-LIM.

### Model runs with less constraint

Due to the difficulty of measuring pelagic ecosystem rates in situ, most LIM studies have a lower ratio of measurement constraints to unknown flows than our base LIM studies. As a result, we conducted tests in which we withheld either single measurements or pairs of measurements. These simulations were conducted by sequentially withholding a single measurement constraint (nitrate uptake, mesozooplankton grazing, or sediment trap-derived export) or pairs of those measurements. Primary productivity was never withheld as a measurement constraint, because it has been measured in every pelagic LIM study that we have encountered.

### Statistical analysis

To compute 95% confidence intervals for derived variables (e.g. trophic level) from MCMC and MCMC+^15^N approaches, we computed the derived variable for each individual solution and took the 95% confidence intervals of this distribution of derived variables. We depict these values as whisker plots showing means, quartiles, and 95% confidence intervals. For an objective comparison of the efficacy with which each LIM approach depicted the underlying “true” ecosystem structure we compared LIM estimates for each of the withheld results in Table 2. These varied ecosystem indices could be similarly calculated for both forward models and the LIM ecosystem reconstructions and together gave a composite snapshot of ecosystem structure. To ensure that each of the 15 indices was given equivalent weight, we first pooled results for each individual index (e.g. all estimates of N_2_ fixation from the four forward model runs and from the 112 LIM runs (4 forward models × 4 approaches × 7 input configurations)). We then used a two-parameter Box Cox transformation (R function boxcoxfit) to normalize this pooled data, subtracted off the mean, and divided by the standard deviation. Thus values for all indices were approximately normal, with a mean of 0 and standard deviation of 1. To determine a composite index that assessed the overall effectiveness with which any LIM model run recovered the underlying “true” values from the forward model, we computed the sum of squared errors (SSE) by subtracted the “true” value from the LIM prediction and summing the square of this value for all 15 indices. To visualize this data in two dimensions we used non-metric multi-dimensional scaling using the mdscale function in Matlab. Dissimilarities used as inputs for mdscale were calculated from Matlab function pdist.

**Table 2:** Model results from the DIAZO and NEMURO models (at steady state).

**Model results used as inputs to inverse model**				
	DIAZO (Coastal)	DIAZO (Mesohaline)	NEMURO (Coastal)	NEMURO (Oligotrophic)
**Net Primary Production (mmol N m**^**-2**^ **d**^**-1**^**)**	2.17	0.70	13.61	2.42
**NO**_**3**_^**-**^ **Uptake (New Production) (mmol N m**^**-2**^ d-1)**	0.72*	0.04*	8.44	0.95
**Mesozooplankton Grazing (mmol N m**^**-2**^ **d**^**-1**^**)**	1.09	0.24	8.11	1.25
**Sediment Trap Export (mmol N m**^**-2**^ **d**^**-1**^**)**	0.42	0.10	7.28	0.69
**Cyanobacteria Biomass (mmol N m**^**-2**^**)**	1.55	1.21	12.12	14.65
**Diatom Biomass (mmol N m**^**-2**^**)**	2.57	1.63	34.58	10.94
**Mesozooplankton Biomass (mmol N m**^**-2**^**)**	6.38	2.14	18.38	17.99
**Temperature (°C)**	28.00	28.00	12.00	14.00
**δ**^**15**^**N exogenous NO**_**3**_^**-**^	7.70	13.25	5.70	5.70
**δ**^**15**^**N Mesozooplankton**	6.10	5.08	7.68	7.23
**δ**^**15**^**N DOM**	4.20	2.95	4.87	4.66
**δ**^**15**^**N Sinking Detritus**	3.98	2.88	4.42	4.82
**Model results withheld from inverse model**				
**New Production (N**_**2**_ **Fixation) (μmol N m**^**-2**^ **d**^**-1**^**)**	3.11	100.48	0	0
**Total N Uptake (mmol N m**^**-2**^ **d**^**-1**^**)**	3.09	0.96	17.16	3.53
**Cyanobacteria Net Primary Production (mmol N m**^**-2**^ **d**^**-1**^**)**	0.48	0.25	2.51	1.33
**Diatom Net Primary Production (mmol N m**^**-2**^ **d**^**-1**^**)**	1.69	0.45	11.10	1.09
**Protozoan Grazing Rate (mmol N m**^**-2**^ **d**^**-1**^**)**	0.73	0.36	0.53	0.74
**Mesozooplankton Carnivory (MES→MES) (mmol N m**^**-2**^ **d**^**-1**^**)**	0	0	1.89	0.24
**Protozoan Carnivory (HNF→MIC) (mmol N m**^**-2**^ **d**^**-1**^**)**	0.11	0.07	0	0
**Mesozooplankton Secondary Production (mmol N m**^**-2**^ **d**^**-1**^**)**	0.30	0.04	1.16	0.26
**Protozoan GGE**	0.3	0.3	0.3	0.3
**Mesozooplankton GGE**	0.3	0.3	0.3	0.3
**Protozoan Trophic Level**	2.12	2.22	2.00	2.00
**Mesozooplankton Trophic Level**	2.15	2.21	2.46	2.37
**Herbivorous Food Chain^1^ (Percent Total NPP)**	0.50	0.34	0.23	0.27
**Multivorous Food Chain^2^ (Percent Total NPP)**	0.34	0.51	0.01	0.16
**Microbial Loop Food Web^3^ (Percent Total NPP)**	0.80	0.83	0.19	0.28
**δ**^**15**^**N Euphotic Zone NO**_**3**_^**-**^	13.25**	11.96**	9.92	8.84
**δ**^**15**^**N NH**_**4**_^**+**^	13.25**	11.96**	12.80	13.60
**δ**^**15**^**N Cyanobacteria**	3.03	1.73	3.91	3.65
**δ**^**15**^**N Diatoms**	3.17	1.59	3.87	3.64
**δ**^**15**^**N Protozoans**	6.17	5.04	6.52	6.26

Upper portion of table shows results that were given as input to the inverse models. Lower portion shows results that were withheld and can be used as comparisons for the inverse models.

^1^Herbivorous Food Chain is proportion of NPP routed through the direct phytoplankton→ mesozooplankton→ higher trophic levels food chain.

^2^Multivorous food chain is proportion of NPP that enters food chains that pass through multiple zooplankton before reaching higher trophic level.

^3^Microbial loop is proportion of NPP that is (implicitly) processed by bacteria through degradation of DOM to NH_4_^+^.

*The DIAZO model has only one dissolved inorganic nitrogen compartment (DIN, rather than NO_3_^-^ and NH_4_^+^), hence new production that would be equivalent to nitrate uptake is calculated from the proportion of exogenous (non-recycled) DIN used by phytoplankton.

**The δ^15^N value of the single DIN pool in DIAZO is given for both NO_3_^-^ and NH_4_^+^.

## Results and discussion

### Forward model results

We used two models (DIAZO and NEMURO), each run twice to simulate different ecosystem conditions, to develop four ecosystem snapshots for use as inputs to the inverse modeling algorithms (Table 2). The DIAZO model was configured to simulate conditions in the coastal and mesohaline region of the Amazon River Plume. In the coastal region, nutrients derived from the Amazon River (0.8 μmol L^-1^ phosphate, 32 μmol L^-1^ silicic acid, and 8.5 μmol L^-1^ nitrate with a riverine δ^15^N value of 7.7) support a moderately productive ecosystem. Diatoms fueled 70% of net primary production and this diatom-dominance was reflected in the zooplankton community structure with mesozooplankton responsible for 60% of total grazing and maintaining a biomass 4 times greater than protistan zooplankton. These mesozooplankton were at a trophic level of 2.15 (reflecting a predominantly diatom diet) and had a δ^15^N value of 6.10.

In the mesohaline region, where diatom-diazotroph assemblages are expected to be common, the DIAZO model predicted substantial N_2_ fixation, with N_2_ fixation supporting 74% of the total new production in the system. Net primary production was reduced relative to the coastal region (0.70 mmol N m^-2^ d^-1^, compared to 2.17 mmol N m^-2^ d^-1^). Cyanobacteria:diatom biomass was more closely balanced in the mesohaline region and a substantial portion of the diatom community was comprised of diatom-diazotroph assemblages. Mesozooplankton had a similar trophic level to that in the coastal region (2.21) although they comprised a lower proportion of total grazing (40%) and had a substantially lower δ^15^N signature of 5.08 reflecting the increased importance of N_2_ fixation in this region.

The NEMURO model was used to simulate plankton communities in the coastal upwelling region of the California Current Ecosystem and the oligotrophic North Pacific Subtropical Gyre. Both systems were supported by upwelled nutrients at a concentration of 10 μmol NO_3_^-^ L^-1^ and 10 μmol Si L^-1^, but the upwelling rates differed substantially (1 m d^-1^ into a 30-m euphotic layer in the coastal region; 0.1 m d^-1^ into a 100-m euphotic layer in the oligotrophic region). The coastal region exhibited the highest net primary productivity (13.6 mmol N m^-2^ d^-1^) and *f*-ratio (new production / total production = 49%) of our model runs. Diatoms were the dominant phytoplankters (65% of biomass; 82% of production) and mesozooplankton were responsible for nearly all grazing (94%). Despite their substantial grazing rates, mesozooplankton had a high trophic level of 2.46, reflecting the prevalence of mesozooplankton carnivory (unlike in the DIAZO model, the NEMURO model contains a predatory mesozooplankton class that can feed on other mesozooplankton).

The offshore oligotrophic NEMURO run had the lowest net primary productivity by volume of any of our model runs (although vertically integrated primary productivity was higher than in the DIAZO model runs, as we assumed a 10-m mixed layer for the Amazon Plume and a 100-m euphotic zone for the oligotrophic subtropical gyre). This low productivity was matched by cyanobacterial dominance of the phytoplankton community (57% of biomass; 55% of production). However, this cyanobacteria production did not translate into an important role for protozoans, which were responsible for 37% of the grazing. This high cyanobacteria / low protozoan condition is determined by the structure of NEMURO, which allows mesozooplankton to graze on both phytoplankton classes, but restricts protozoans to consuming only cyanobacteria. Mesozooplankton had a lower trophic level and δ^15^N than in the coastal upwelling region, reflecting reduced rates of carnivory.

### Inverse model ecosystem reconstructions

**Fig 3:**
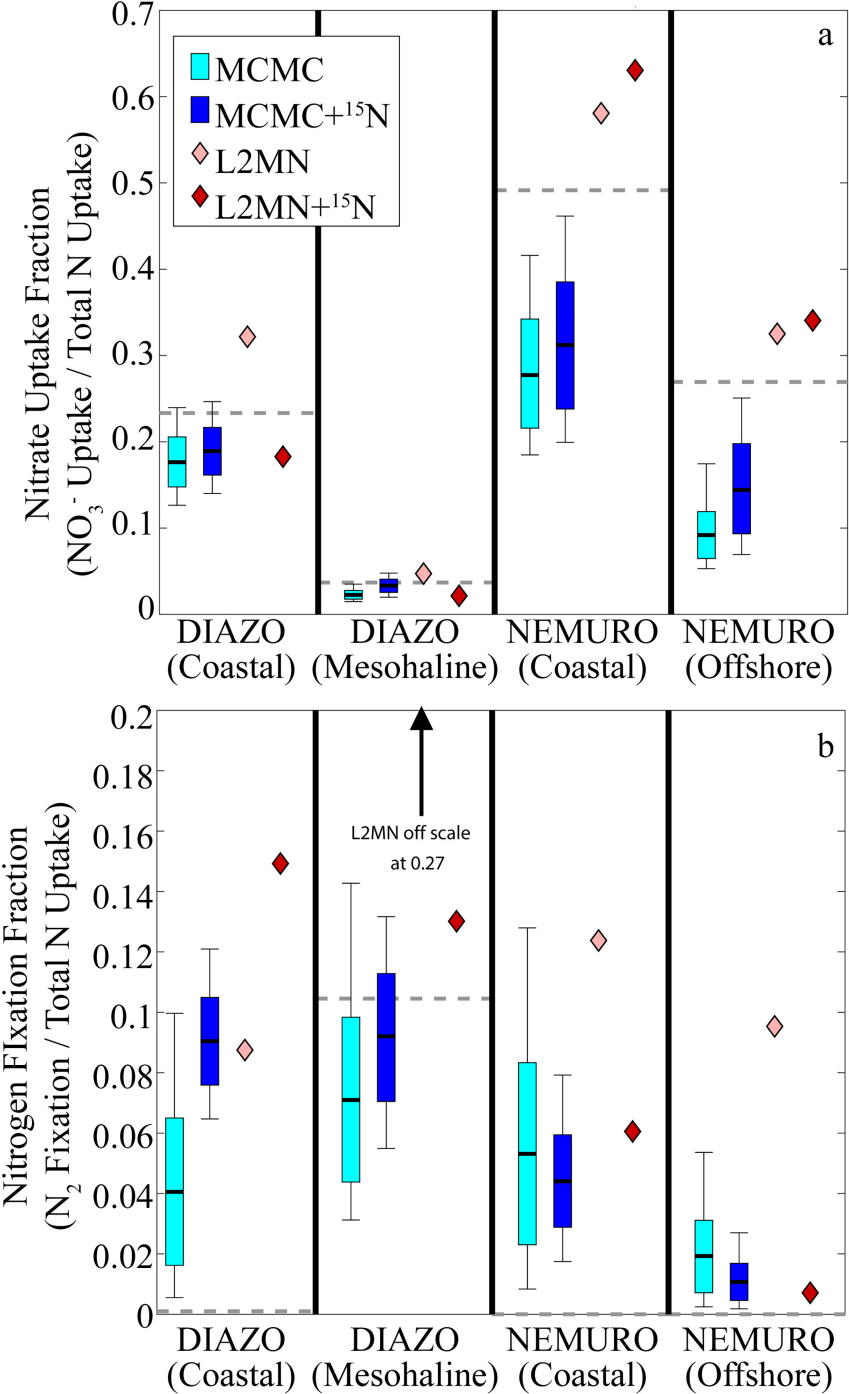
LIM NO_3_^-^ Uptake (a) and N_2_ Fixation (b). Both plots show fraction of total phytoplankton nitrogen supplied by respective process. Light and dark box plots show 95% confidence intervals, quartiles, and mean for MCMC and MCMC+^15^N, respectively. Light and dark diamonds show values determined by L2MN and L2MN+^15^N, respectively. Dashed gray line shows “true” value from the forward model run.

Results from the forward model run (primary production, nitrate uptake, mesozooplankton grazing, cyanobacteria biomass, diatom biomass, mesozooplankton biomass, and the δ^15^N signatures of exogenous NO_3_^-^, mesozooplankton, DOM, and sinking detritus) were used to force LIM simulations using the L2MN, L2MN+^15^N, MCMC, and MCMC+^15^N approaches. To assess the accuracy of the nutrient dynamics in each of the LIM simulations, we compared the ratio of NO_3_^-^ uptake to total nitrogen uptake and N_2_ fixation to total nitrogen uptake (Fig 3, S1 Fig). A distinct difference was clear between the L_2_ minimum norm approaches and the Monte Carlo approaches, with both MCMC approaches consistently underestimating the ratio of percent nitrogen taken up as new nitrate and the L2MN approaches typically overestimating nitrate uptake percentage. However, the addition of ^15^N information consistently improved the MCMC+^15^N approach relative to the standard MCMC approach. The mean percent error decreased from a 43% underestimate with the MCMC approach to a 27% underestimate with the MCMC+^15^N approach. When comparing the LIM approaches’ recovery of N_2_ fixation rates all four approaches overestimated N_2_ fixation for the DIAZO coastal run and both NEMURO model runs (which was unsurprising since the NEMURO model does not allow N_2_ fixation). However, the L2MN approach (without ^15^N) typically performed worse than the other three approaches, estimating that N_2_ fixation was always between 8 and 13% of total nitrogen uptake (when the “true” value was negligible), while the other three approaches were comparable and suggested fractions of 1% - 9% (except for the L2MN+^15^N approach with the DIAZO coastal model run, which predicted 15%). For the DIAZO mesohaline run, the “true” value from the forward model was 10% and the MCMC approaches slightly underestimated this value (7% for MCMC; 9% for MCMC+^15^N), while the L2MN+^15^N approach slightly overestimated it at 13% and the L2MN approach substantially overestimated it at 27%.

**Fig 4:**
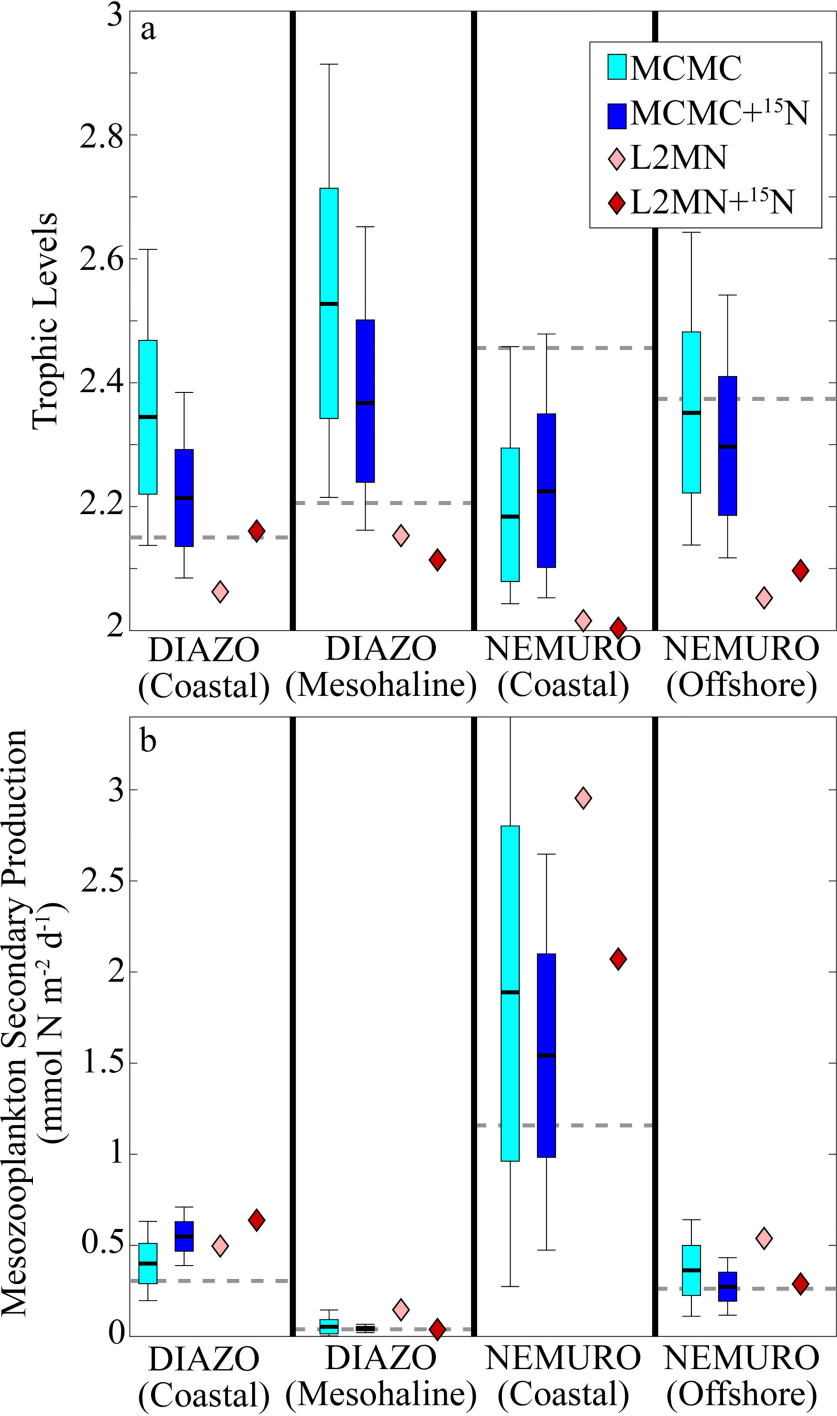
LIM Mesozooplankton trophic level (a) and secondary production (b). LIM secondary production is the flow from MES to unmodeled higher trophic levels. Light and dark box plots show 95% confidence intervals, quartiles, and mean for MCMC and MCMC+^15^N, respectively. Light and dark diamonds show values determined by L2MN and L2MN+^15^N, respectively. Dashed gray line shows “true” value from the forward model run.

To determine the ability of the LIM approaches to reconstruct grazer dynamics, we compared the forward model values to trophic level and secondary production estimates from the LIM models (Fig 4, S2 Fig). Both MCMC approaches did a reasonable job of recovering mesozooplankton trophic levels for all model simulations (95% confidence intervals consistently bracketed the “true” values), although they were often high or low by ~0.2 trophic levels. However, the L2MN approaches were biased low, particularly with the NEMURO model, for which they predicted trophic levels ranging from 2.01–2.12, while the “true” values were 2.46 and 2.37. For mesozooplankton secondary production (which we define herein as the amount of mesozooplankton production that was consumed by higher trophic levels), we found that the MCMC, MCMC+^15^N, and L2MN+^15^N approaches did a good job of recovering the “true” values for the oligotrophic ecosystem states, but were biased slightly high for the coastal ecosystem states. By contrast, the standard L2MN approach consistently overestimated secondary production, at times predicting values that were greater than three times the expected value.

### Inverse model performance with less constraint

The LIM structure used here is relatively simple and well-constrained compared to many inverse models that include greater taxonomic complexity amongst the plankton, higher trophic level compartments, or multi-layer ecosystems (e.g. [47, 48, 58]). As a result, the rank parameter for our LIM structure (which denotes the number of linearly independent equations in Eqs 1 and 3, and is equal to the number of unknown flows minus the number of constraining equations) is much lower than for many published ecosystem models. For comparison, the rank of the base L2MN structure of our model (35 ecosystem unknowns, 9 mass balance constraints, 4 measurement constraints) is 22, while the Antarctic ecosystem LIM of Sailley et al. [58] contained 48 unknowns, 10 mass balance constraints and only 2 measurement constraints (rank = 36) and the Equatorial Pacific model with size-fractionated detritus of Stukel and Landry [4] contained 62 unknowns, 12 mass balance constraints, and 8 measurement constraints (rank = 42). To determine the efficacy of the 4 LIM approaches when the system has less constraint, we recomputed the solutions while sequentially withholding one or two of our measurement constraints (with the exception of NPP, which has been used as an input for every pelagic ecosystem LIM that we have seen), thus yielding 6 additional LIM solutions for each approach (3 with a single measurement withheld, 3 with pairs of measurements withheld).

**Fig 5:**
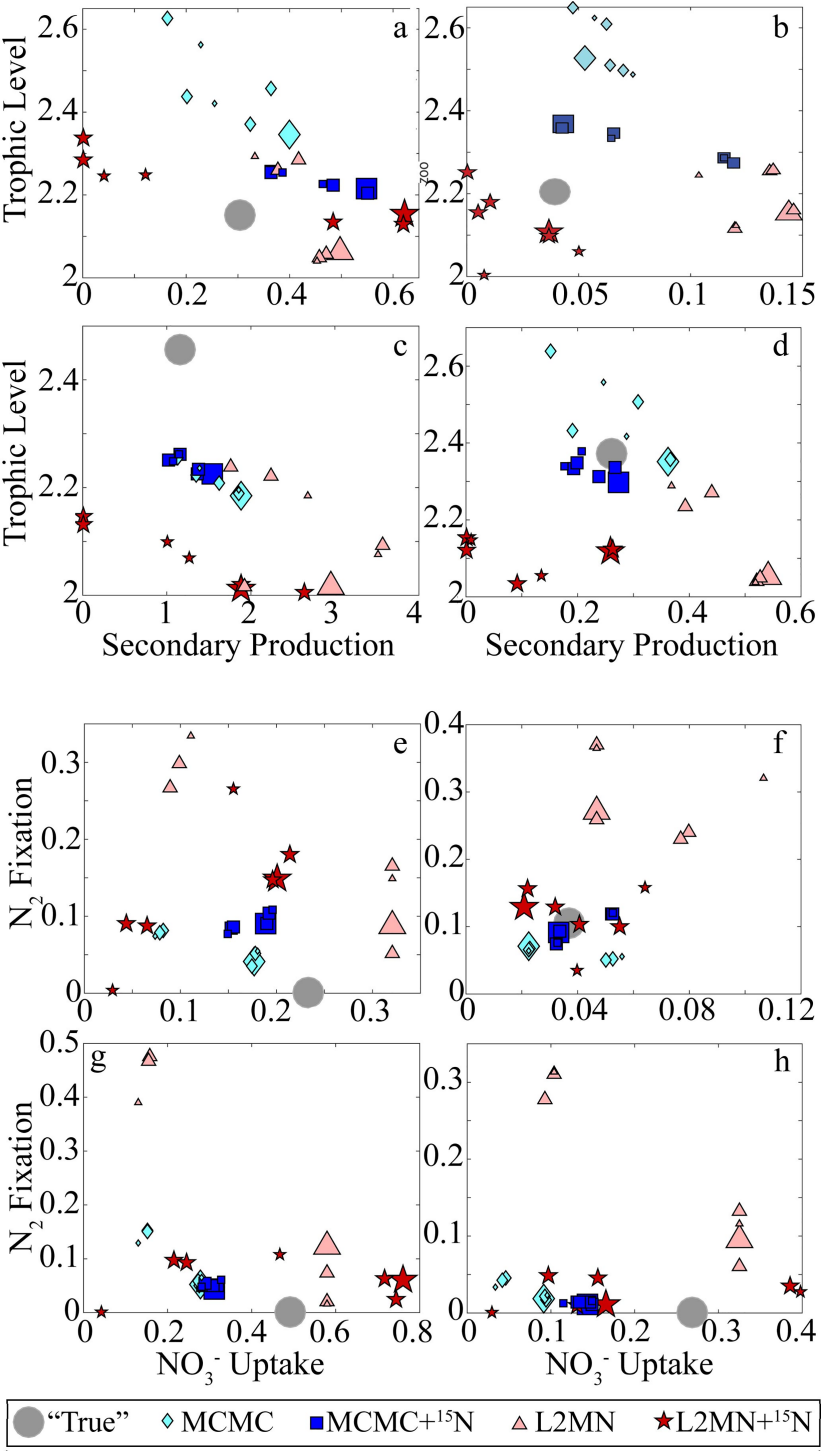
LIM accuracy when input measurements are withheld. Panels a-d show mesozooplankton secondary production (mmol N m^-2^ d^-1^, x-axis) against mesozooplankton trophic level (y-axis). Panels e-g show NO_3_^-^ uptake (fraction of total N uptake, x-axis) against N_2_ fixation (fraction of total N uptake, y-axis). Gray circles indicate the “true” values from the forward models. Other symbols are MCMC (square), MCMC+^15^N (diamond), L2MN (triangle), and L2MN+^15^N (star). Symbol size reflects the number of measurements withheld as inputs for the inverse model (large is no measurements withheld, medium is one measurement withheld, small is two measurements withheld). a,e) DIAZO Coastal; b,f) DIAZO Mesohaline; c,g) NEMURO Coastal; d,h) NEMURO Offshore.

When assessing mesozooplankton dynamics (trophic level and secondary production), the MCMC+^15^N model showed relatively little degradation in accuracy as the model became more underconstrained (Fig 5A–5D). In fact, for the two coastal simulations, trophic level estimates did not change substantially when measurements were withheld, but secondary production estimates decreased to more closely match the “true” value determined by the forward model run. For the mesohaline and offshore runs, the model performed slightly more poorly on average when measurements were withheld. Similar results were found when comparing nitrogen dynamics estimated with measurements withheld (Fig 5E–5H); results obtained by the MCMC+^15^N approach were relatively insensitive to which measurements were used as inputs to the model. By contrast, the offset between the MCMC estimate and the “true” value tended to be a bit larger when more measurements were withheld, while solution sets from both L2MN approaches exhibited strong sensitivity to which measurements were used as inputs (error often switched from over- to under-estimates, or vice versa, depending on which measurements were withheld).

**Fig 6:**
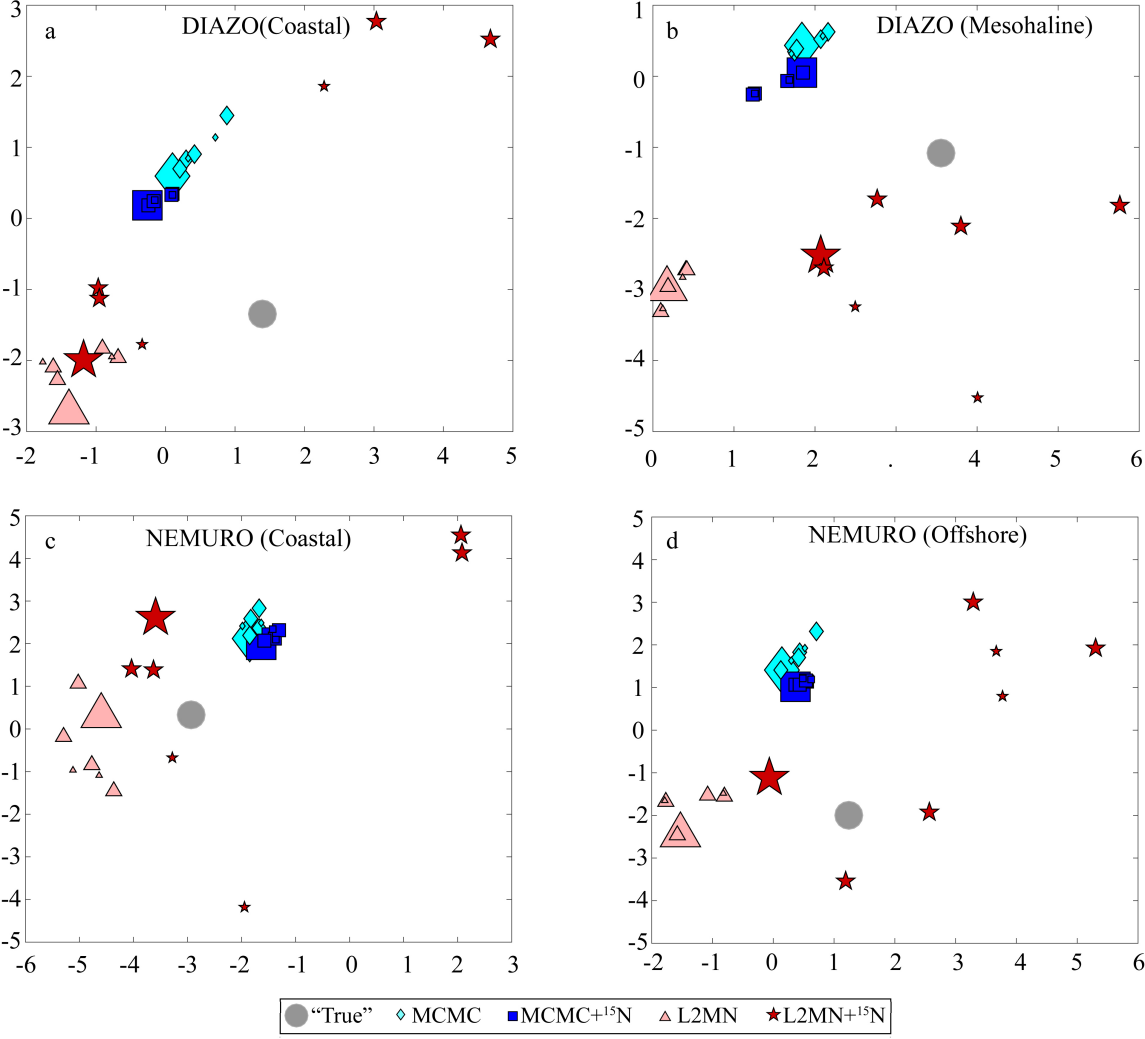
Non-metric multi-dimensional scaling (NMDS) plot showing model variability with respect to the 15 model results withheld from the inverse model in Table 2 (excluding δ^15^N values). The minimized stress of the NMDS analysis was 0.16.

For a holistic comparison of the efficacy with which each LIM approach depicted the underlying “true” ecosystem structure we compared LIM estimates for each of the withheld results in Table 2. We computed the sum of squared errors (SSE) for each LIM model run with respect to the “true” values for each of these indices. This composite value reflects how well the LIM ecosystem structure captures the overall value. With respect to SSE, the MCMC+^15^N approach performed better than the standard MCMC approach for 27 of the 28 model-measurement input pairings and was the best of the four approaches for 23 of the 28 scenarios. Compared to the other approaches, the MCMC+^15^N approach also showed less deterioration in accuracy when measurements were withheld. With all input measurements included, the median SSE for the MCMC+^15^N approach was 20.7 while it was 20.8 with one measurement withheld and 20.2 with two measurements withheld. The relative insensitivity of the MCMC+^15^N approach to removal of measurements is also clear when the results are plotted using non-metric multi-dimensional scaling (NMDS, Fig 6). While the MCMC approaches tended to cluster together, the results from the L2MN approaches were highly sensitive to which measurements were used as inputs and often produced results on opposite sides of the NMDS plot. These highly divergent results arise from the L2MN approaches’ tendency to select solution sets that lie on the edge of the possible solution space (e.g. gross growth efficiency, which can vary from 0.1 to 0.4 is usually placed at exactly 0.1 or 0.4).

### δ^15^N values

**Fig 7:**
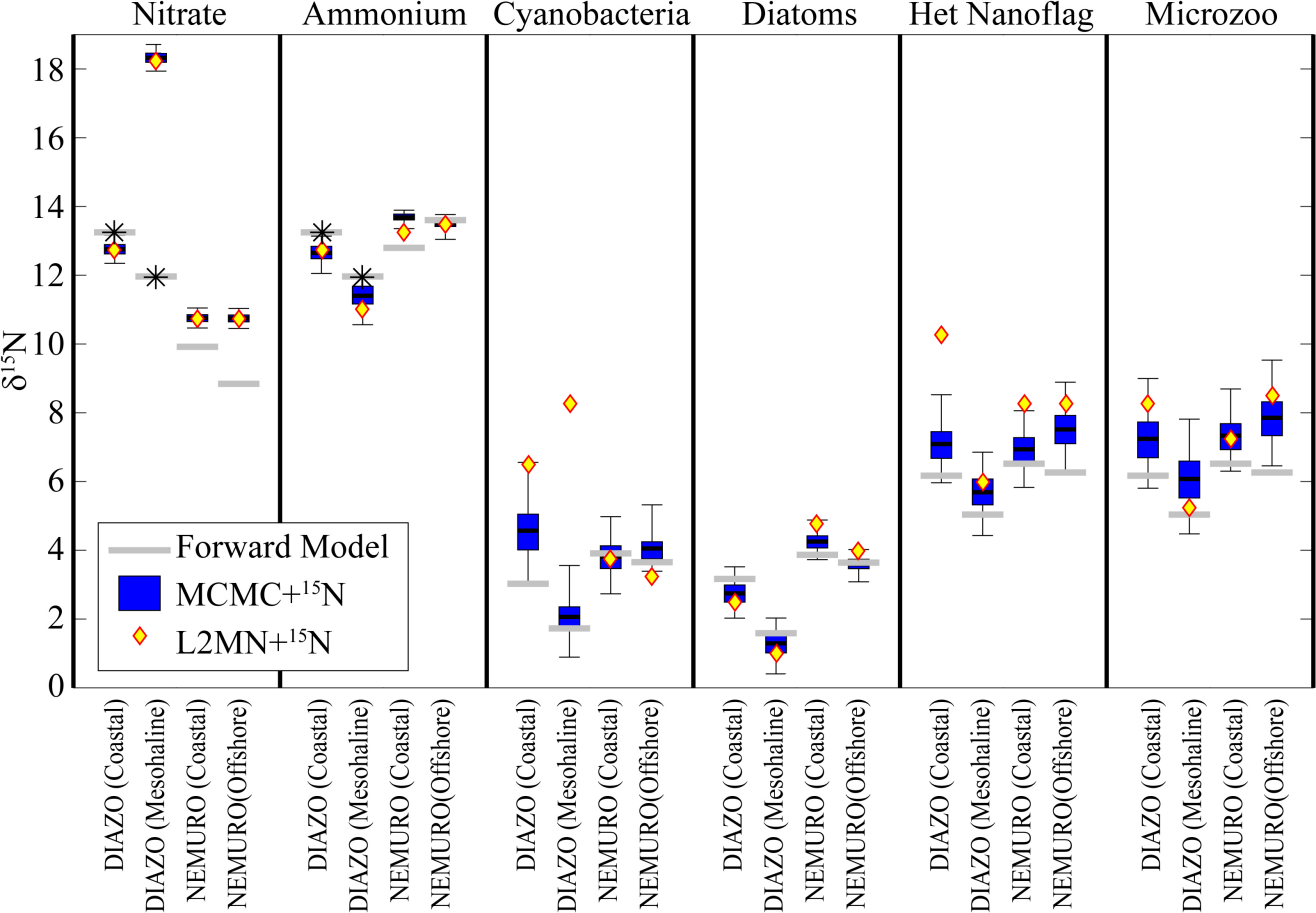
LIM model estimated δ^15^N values. Box plots show 95% confidence intervals, quartiles, and mean for MCMC+^15^N. Diamonds show values determined by L2MN+^15^N. Dashed gray line shows “true” value from the forward model run. * indicates that the DIAZO model had only one dissolved inorganic nutrient pool, while the LIM models had two.

The MCMC+^15^N approach also did an excellent job of recovering the δ^15^N signatures of model compartments (Fig 7). Most of the time, the model 95% confidence intervals bracketed the actual values and a Type 1 linear regression of the MCMC+^15^N predicted values regressed against the “true” values was statistically significant (p<<0.001) with a slope of 1.05, an intercept of 0.39, and an r^2^ value of 0.90. The only major discrepancy between the MCMC+^15^N values and the “true” values arose with the δ^15^N value of NO_3_^-^ for the DIAZO model. This misfit was not unexpected as the DIAZO model has only one dissolved inorganic nitrogen state variable (the δ^15^N value of which was used for the “true” value for both NO_3_^-^ and NH_4_^+^). In most instances, the L2MN+^15^N also did a reasonable job of recovering most δ^15^N values. However, occasionally estimates generated by this approach were far from the “true” values, particularly when dealing with compartments through which the L2MN+^15^N approach predicted relatively little energy throughput. Because of these occasional large misfits, the r^2^ value of a linear regression was lower (0.76).

### Considerations for use with in situ data sets

In this study, we used simulated datasets from four configurations of dynamical (forward) models to test the efficacy with which static, mass-balanced LIM approaches recover ecosystem structure when only given inputs that are typically measured in situ. This approach has proven a powerful way to assess investigate LIM methodology [34, 59], although it is not without its limitations due to the inherent differences between dynamic simulations and steady-state LIM models [60]. To alleviate these issues, our model simulations were run to steady state with constant physical forcing (i.e. constant upwelling in NEMURO simulations; constant river input in DIAZO simulations). The ocean is seldom at a true steady state, suggesting that it could be fruitful for future studies to use results derived from non-steady state, three-dimensional, coupled biogeochemical models as inputs for assessing LIM accuracy. However, prior work has suggested that LIM results based on transient states are as accurate as those derived from true steady-state conditions [34].

When using LIM data assimilation techniques it is important to consider the inherent biases of the L2MN and MCMC approaches. Well known biases associated with the L2MN approach have been assessed in other manuscripts [27, 29, 30] and are related to the L2MN approach’s goal of minimizing total flow through the ecosystem. In our simulations, this was apparent in the L2MN approach’s attempt to minimize recycled production (minimizing NH_4_^+^ production by multiple compartments while maximizing N_2_ fixation) and minimize the number of trophic steps through the zooplankton (thus typically underestimating mesozooplankton trophic level and overestimating secondary production). The MCMC approach, however, has subtle biases of its own that must be considered. Specifically, a greater portion of the solution space tends to exist at high total system throughput (i.e. sum of all flows) than at low total system throughput. Thus the MCMC mean solutions (which average across all possible solution sets) tend to be biased towards solutions that increase total flow, often by including many trophic steps and enhanced recycling.

In addition to these biases, LIM model results can be affected by the assumed ecosystem structure used to construct the model. For instance, increased aggregation of functional groups (i.e. inclusion of less compartments) was shown to decrease LIM model accuracy in a tidal system [61]. In pelagic systems, even splitting detritus into three size-structured detritus compartments can substantially impact the relative contribution of different phytoplankton groups to total export [4]. Unfortunately, the appropriate level of aggregation and the true ecosystem structure are seldom known a priori and must be estimated by the investigator. In this way, our decision to use different ecosystem structure for the LIM and the two forward models allows us to simulate the difficulties found in the field. For instance, the LIM model allows diazotrophy, which is absent from the NEMURO forward model. Thus the LIM model consistently overestimates the “true” rate of N_2_ fixation (zero). An important result of our study was that the inclusion of ^15^N data improved the LIM model’s ability to reconstruct the “true” values despite these differences in model structure.

Regardless of which approach is used (MCMC or L2MN), the inclusion of ^15^N data provides additional constraint on the system. This agrees with the finding of Vézina and Pahlow [34] that inverse approaches using multiple currencies (e.g. C and N, or N and ^15^N) were more accurate than approaches using only a single currency. When applied to natural pelagic ecosystems that are usually highly underconstrained due to the difficulty of measuring planktonic rates in situ, we expect that both ^15^N approaches will outperform the results of LIM models without this additional data source. However, although our forward models and LIM approaches assumed the same known isotopic fractionation factors, in situ fractionation factors should be assumed to have some uncertainty to them. Indeed, our understanding of taxonomic diversity in fractionation processes is still evolving. For instance, the isotopic fractionation coefficients for zooplankton (ε_exc_ and ε_eg_) together control the trophic enrichment factor (TEF) of consumers, which has typically been assumed to be in the range of 3–3.5 for a diverse suite of organisms. However, recent evidence [62, 63] suggests that the TEF of protozoans is much lower (less than 1), thus necessitating lower values for ε_exc_ and ε_eg_. If accurate 95% confidence intervals on δ^15^N values are required, it is thus likely necessary to incorporate uncertainty in isotopic fractionation coefficients.

When used with in situ data sets, the MCMC+^15^N approach can be easily adapted to incorporate variable stoichiometric data (with or without δ^15^N data) if such datatypes are available. It would be trivial to replace ∂_K_ and ∂_U_ (the vectors containing the known and unknown/varying δ^15^N values, respectively) with vectors including C:N, N:P, or δ^13^C data, each of which could be used to update the approximate equations stored in matrix A. In this way, our approach is best seen as a flexible tool that can be used to assimilate diverse available datasets into a constrained estimate of ecosystem structure.

## APPENDIX 1 –approximate equalities

Input measurements used for all LIM approaches

NFixDTM + NO3→DTM + NH4→DTM − DTM→DOM + NFixCYA + NO3→CYA + NH4→CYA − CYA→DOM = NPP

DTM→MES = MesozooGrazing

NO3→DTM + NO3→CYA = NitrateUptake

DET→Sink = SedimentTrapExport

^15^N Mass balance equations used for L2MN+^15^N and MCMC+^15^N

R_upno3_×EXT→NO3 − (R_NO3_×α_no3_)×NO3→CYA − (R_NO3_×α_no3_)×NO3→DTM = 0

−(R_nh4_×α_nh4_)×NH4→DTM − (R_nh4_×α_nh4_)×NH4→CYA + (R_hnf_×α_exc_)×HNF→NH4 + (R_mic_×α_exc_)×MIC→NH4 + (R_mes_×α_exc_)×MES→NH4 + (R_DOM_×α_sol_)×DOM→NH4 = 0

R_nfix_×NFixCYA + (R_NO3_×α_no3_)×NO3→CYA + (R_nh4_×α_nh4_)×NH4→CYA − R_cya_×CYA→HNF − R_cya_×CYA→MIC − R_cya_×CYA→DET − R_cya_×CYA→DOM = 0

R_nfix_×NFixDTM + (R_NO3_×α_no3_)×NO3→DTM + (R_nh4_×α_nh4_)×NH4→DTM–R_dtm_×DTM→MIC–R_dtm_×DTM→MES–R_dtm_×DTM→DET–R_dtm_×DTM→DOM = 0

R_cya_×CYA→HNF + R_det_×DET→HNF–R_hnf_×HNF→MIC–R_hnf_×HNF→MES–(R_hnf_×α_exc_)×HNF→NH4 –(R_hnf_×α_eg_)×HNF→DET–(R_hnf_×α_exc_)×HNF→DOM = 0

R_cya_×CYA→MIC + R_dtm_×DTM→MIC + R_hnf_×HNF→MIC + R_det_×DET→MIC–R_mic_×MIC→MES–(R_mic_×α_exc_)×MIC→NH4 –(R_mic_×α_eg_)×MIC→DET–(R_mic_×α_exc_)×MIC→DOM = 0

R_dtm_×DTM→MES + R_hnf_×HNF→MES + R_mic_×MIC→MES + R_det_×DET→MES–R_mes_×MES→HTL–(R_mes_×α_exc_)×MES→NH4 –(R_mes_×α_eg_)×MES→DET–(R_mes_×α_exc_)×MES→DOM = 0

R_dtm_×DTM→DET + R_cya_×CYA→DET + (R_hnf_×α_eg_)×HNF→DET + (R_mic_×α_eg_)×MIC→DET + (R_mes_×α_eg_)×MES→DET–R_det_×DET→HNF–R_det_×DET→MIC–R_det_×DET→MES–(R_det_×α_sol_)×DET→DOM–R_det_×DET→Sink = 0

R_dtm_×DTM→DOM + R_cya_×CYA→DOM + (R_hnf_×α_exc_)×HNF→DOM + (R_mic_×α_exc_)×MIC→DOM + (R_mes_×α_exc_)×MES→DOM + (R_det_×α_sol_)×DET→DOM–(R_dom_×α_sol_)×DOM→NH4 = 0

R_upno3_×EXT→NO3 + R_nfix_×NFixCYA + R_nfix_×NFixDTM–R_det_×DET→Sink–R_mes_×MES→HTL = 0

In all equations R_x_ refers to the ^15^N:^14^N isotopic ratio of compartment x, which is computed from δ^15^N values using the equation R_x_ = δ^15^N_x_ × R_N2_ / 1000 + R_N2_, where R_N2_ is the ^15^N:^14^N isotopic ratio of atmospheric dinitrogen gas. In all equations α_y_ refers to the isotopic fractionation coefficient for process y and is calculated from the isotopic fractionation factor (ε_y_) for process y according to the equation α_y_ = exp(ε_y_/1000). Fractionation factors used in this study were taken from Yoshikawa et al. [41] and had values of ε_NO3_ = -5‰, ε_NH4_ = -10‰, ε_exc_ = -5‰, ε_eg_ = -2‰, ε_rem_ = -1‰.

## Supporting information

S1 Supplementary Text A detailed description of our implementation of the MCMC+^15^N LIM method. (PDF)

S1 Fig Histograms of nitrate uptake fraction (x-axis) and nitrogen fixation fraction (y-axis) and a scatter plot of nitrogen fixation fraction against nitrate uptake fraction as computed by the MCMC+^15^N approach. For comparison, the MCMC approach mean value is shown in cyan diamond (with 95% confidence interval) and L2MN and L2MN+^15^N values are shown in pink and dark red, respectively.
(JPG)

S2 Fig Histograms of mesozooplankton secondary production (x-axis) and mesozooplankton trophic level (y-axis) and a scatter plot of trophic level against secondary production as computed by the MCMC+^15^N approach. For comparison, the MCMC approach mean value is shown in cyan diamond (with 95% confidence interval) and L2MN and L2MN+^15^N values are shown in pink and dark red, respectively.
(JPG)
