# Supplementary material for: A new approach for incorporating 15N isotopic data into linear inverse ecosystem models with Markov Chain Monte Carlo sampling
Source: PLoS One. 2018 Jun 18;13(6):e0199123. doi: 10.1371/journal.pone.0199123 (PMC6005467; doi:10.1371/journal.pone.0199123)
Supplement: S1 Supplementary Text — (PDF) [file pone.0199123.s001.pdf]

Supplementary material for:

# A new approach for incorporating $^{15}N$ isotopic data into linear inverse ecosystem models with Markov Chain Monte Carlo sampling

Michael R. Stukel, Moira Décima, Thomas B. Kelly

## Supplementary Text

*Inverse methodology equations* – In addition to the approximate equalities shown in appendix 1, the L2MN, MCMC, L2MN+$^{15}N$, and MCMC+$^{15}N$ approaches all solved the following mass balance equality constraints and inequality constraints drawn from *a priori* knowledge about ecosystem structure and physiological constraints.

<u>Mass balance constraints (Ax=b):</u>

EXT→NO3 − NO3→CYA − NO3→DTM = 0

−NH4→DTM − NH4→CYA + HNF→NH4 + MIC→NH4 + MES→NH4 + DOM→NH4 = 0

NFixCYA + NO3→CYA + NH4→CYA − CYA→HNF − CYA→MIC − CYA→DET − CYA→DOM = 0

NFixDTM + NO3→DTM + NH4→DTM –DTM→MIC – DTM→MES – DTM→DET – DTM→DOM = 0

CYA→HNF + DET→HNF – HNF→MIC – HNF→MES – HNF→NH4 –HNF→DET – HNF→DOM = 0

CYA→MIC + DTM→MIC + HNF→MIC + DET→MIC – MIC→MES – MIC→NH4 – MIC→DET – MIC→DOM = 0

DTM→MES + HNF→MES + MIC→MES + DET→MES – MES→HTL – MES→NH4 – MES→DET – MES→DOM = 0

DTM→DET + CYA→DET + HNF→DET + MIC→DET + MES→DET – DET→HNF – DET→MIC – DET→MES – DET→DOM – DET→Sink = 0

DTM→DOM + CYA→DOM + HNF→DOM + MIC→DOM + MES→DOM + DET→DOM – DOM→NH4 = 0

<u>Inequality constraints (Gx≥h):</u>

-0.2×CYA→HNF + HNF→NH4 – 0.2×DET→MIC ≥ 0

-0.2×DTM→MIC – 0.2×CYA→MIC – 0.2×HNF→MIC + MIC→NH4 – 0.2×DET→MIC ≥ 0

– HNF→NH4 – MIC→NH4 ≥ -1.7×$W_1^{-0.25} \times e^{0.0693\times(T\text{-}20)}$×*ProtozoanBiomass*

-0.2×DTM→MES – 0.2×HNF→MES – 0.2×MIC→MES + MES→NH4 – 0.2×DET→MES – 0.2×MesozooCarn ≥ 0

– MES→NH4 ≥ -1.7×$W_2^{-0.25} \times e^{0.0693\times(T\text{-}20)}$×*MesozooBiomass*

DTM→DOM ≥ 0.02 × *DiatomBiomass*

– DTM→DOM ≥ -0.55× *DiatomBiomass*

CYA→DOM ≥ 0.02 × *CyanoBiomass*

– CYA→DOM ≥ -0.55× *CyanoBiomass*

-0.1×CYA→HNF + HNF→DOM – 0.1×DET→HNF ≥ 0

HNF→NH4 – HNF→DOM ≥ 0

-0.1×DTM→MES – 0.1×HNF→MES – 0.1×MIC→MES + MES→DOM – 0.1×DET→MES – 0.1×MES→MES ≥ 0

MES→NH4 – MES→DOM ≥ 0

0.5×CYA→HNF – HNF→DET + 0.5×DET→HNF ≥ 0

-0.1×CYA→HNF + HNF→DET – 0.1×DET→HNF ≥ 0

0.5×DTM→MIC + 0.5×CYA→MIC + 0.5×HNF→MIC – MIC→DET + 0.5×DET→MIC ≥ 0

-0.1×DTM→MIC – 0.1×CYA→MIC – 0.1×HNF→MIC + MIC→DET – 0.1×DET→MIC ≥ 0

0.5×DTM→MES + 0.5×HNF→MES + 0.5×MIC→MES – MES→DET + 0.5×DET→MES + 0.5× MES→MES ≥ 0

-0.1×DTM→MES – 0.1×HNF→MES – 0.1×MIC→MES + MES→DET – 0.1×DET→MES – 0.1× MES→MES ≥ 0

0.9×CYA→HNF – HNF→NH4 – HNF→DET – HNF→DOM + 0.9×DET→HNF ≥ 0

-0.6×CYA→HNF + HNF→NH4 + HNF→DET + HNF→DOM – 0.6×DET→HNF ≥ 0

0.9×DTM→MIC + 0.9×CYA→MIC + 0.9×HNF→MIC – MIC→NH4 – MIC→DET – MIC→DOM + 0.9×DET→MIC ≥ 0

-0.6×DTM→MIC – 0.6×CYA→MIC – 0.6×HNF→MIC + MIC→NH4 + MIC→DET + MIC→DOM – 0.6×DET→MIC ≥ 0

0.9×DTM→MES + 0.9×HNF→MES + 0.9×MIC→MES – MES→NH4 – MES→DET – MES→DOM + 0.9×DET→MES + 0.9× MES→MES ≥ 0

-0.6×DTM→MES – 0.6×HNF→MES – 0.6×MIC→MES + MES→NH4 + MES→DET + MES→DOM – 0.6×DET→MES – 0.6× MES→MES ≥ 0

T is the temperature (°C). $W_1$ is the average mass of a protozoan (7.5) and $W_2$ is the average mass of a mesozooplankton ($3.8\times10^6$).

*Inverse methodology details (MCMC)* – The MCMC approach uses the xsample function with mirror algorithm found in R package limSolve [50,51]. This approach finds solutions to the system of equations and inequalities Ex=f (Eq. 1) and Gx≥h (Eq. 3) that also approximate the equations Ax≈b (Eq. 2). The approach begins by using a linear transformation such that all solutions to the equality Ex=f can be rewritten as:

$$= {}_0 + Zq \qquad \text{(S1)}$$

where Z is an orthonormal matrix that is calculated by the singular value decomposition of the matrix E and $x_0$ is an initial solution that solves the equality and inequality constraints. We used the L2MN solution (found using the R function lsei) as our initial solution. The MCMC approach then follows an iterative approach to find new solutions through a constrained random walk through the solution space:

1) Given solution $x_n = x_0 + Zq_n$ a new solution is found by a random jump such that $q_{n+1} = q_n + jmp \times R_n$, where jmp is a pre-selected jump length and $R_n$ is a vector with the same length as $q_n$ that is drawn from a random normal distribution.
2) The mirror algorithm is used to reflect the solution $q_{n+1}$ off hyper planes defined by the inequalities (Eq. 3), to ensure that the solution derived from $q_{n+1}$ will also satisfy the inequality constraints.
3) $x_{n*}$ is then defined such that $x_{n*} = x_0 + Zq_{n+1}$ and the probability of $x_{n*}$ and $x_n$ with respect to Eq. 2 are calculated: ($p(x)=e^{-1/2\sigma\text{-}2(Ax-b)'(Ax-b)}$).
4) If $p(x_{n*}) > p(x_n)$, then $x_{n*}$ is accepted as a solution and appended to the matrix of solutions as $x_{n+1}$ and the process is repeated from step 1.
5) Otherwise, a random number (r) is drawn from the uniform distribution from 0 to 1. If $r \le p(x_{n*})/p(x_n)$, then $x_{n*}$ is accepted as a solution and appended to the matrix of solutions as $x_{n+1}$, and the process is repeated from step 1.
6) Else, $x_{n*}$ is rejected as a solution and the process is repeated from $x_n$ at step 1.

This process generates a series of solution vectors that satisfy the equality (Eq. 1) and inequality (Eq. 3) constraints while approximately satisfying the approximate equalities (Eq. 2). More

details can be found in Van den Meersche et al. [51], Soetaert and van Oevelen [64], and van Oevelen et al. [2]. We implemented xsample using a jump length (jmp) that was chosen separately for each model run to allow acceptance rates that were typically in the range of 10%. Each MCMC solution was preceded by a burn-in period equal to 20% of the total run length. This burn-in period was used to ensure that the overall solution was not influenced by the arbitrary choice of $x_0$. To allow for rapid dispersal from the initial location ($x_0$ = L2MN solution), for the beginning of the burn-in period we used a standard deviation ($\sigma$) for the approximate equalities (Eq. 2) equal to 10 times the $\sigma$ that would be used throughout the rest of the random walk. Random walk length was at least 100 million, but was adjusted to ensure that for all unknowns (flows) the difference between the mean of the first half of the random walk and the mean of the second half of the random walk was less than 5%. To minimize performance issues associated with memory constraints, we only stored every 10,000$^{th}$ solution vector.

*Inverse methodology details (MCMC+$^{15}$N)* – Our novel MCMC+$^{15}$N LIM solution scheme built off the standard MCMC approach of Van den Meersche et al. [51] as explained above, but included additional approximate equations (Appendix 1) that codify mass balance constraints on the flow of $^{15}$N through the ecosystem. Since the $\delta^{15}$N values of several compartments in the ecosystem were unknown this required simultaneous estimation of these $\delta^{15}$N values during the random walk exploration of the solution space. Our approach used the mean solution to the standard MCMC approach as a starting point and an initial guess of 0 for all the unknown $\delta^{15}$N values (i.e. all $\delta^{15}$N values equal to the $\delta^{15}$N of atmospheric $N_2$). This initial guess is stored in vector $\partial_{U,0}$ while the known $\delta^{15}$N values of compartments for which isotopic values were measured is stored in vector $\partial_K$. Additional solution vectors were determined using the following approach:

1) Given solution $x_n = x_0 + Zq_n$ a new solution is found by a random jump such that $q_{n+1} = q_n + jmp \times R_n$, where jmp is a pre-selected jump length and $R_n$ is a vector with the same length as $q_n$ that is drawn from a random normal distribution.
2) The mirror algorithm is used to reflect the solution $q_{n+1}$ off hyper planes defined by the inequalities (Eq. 3), to ensure that the solution derived from $q_{n+1}$ will also satisfy the inequality constraints.
3) Given previous solution $\delta^{15}$N values stored in vector $\partial_{U,n}$, a new set of $\delta^{15}$N values is selected by a random jump such that $\partial_{U,n*} = q_n + jmp_{15} \times R_n$, where $jmp_{15}$ is a pre-selected jump length and $R_n$ is a vector with the same length as $\partial_n$ that is drawn from a random normal distribution.
4) Since matrix A from Eq. 2 is a function of $\partial_U$ and $\partial_K$ (see Appendix 1), we then call a function written in R (ResetRN15) to re-calculate $A(\partial_{U,n*},\partial_K)$.
5) $x_{n*}$ is then defined such that $x_{n*} = x_0 + Zq_{n+1}$ and the probability of $x_{n*}$ and $x_n$ with respect to Eq. 2 are calculated: $(p(x)=e^{-1/2\sigma\text{-}2(Ax-b)'(Ax-b)})$, where A is a function of $\partial_{U,n}$ and $\partial_K$ or $\partial_{U,n*}$ and $\partial_K$.

6) If $p(x_{n*},A_{n*}) > p(x_n,A_n)$, then $x_{n*}$ is accepted as a solution and appended to the matrix of solutions as $x_{n+1}$, $\partial_{U,n*}$ is accepted as a solution and appended to the matrix of solutions as $\partial_{U,n+1}$, and the process is repeated from step 1.
7) Otherwise, a random number (r) is drawn from the uniform distribution from 0 to 1. If $r \leq p(x_{n*},A_{n*})/p(x_n,A_n)$, then $x_{n*}$ is accepted as a solution and appended to the matrix of solutions as $x_{n+1}$, $\partial_{U,n*}$ is accepted as a solution and appended to the matrix of solutions as $\partial_{U,n+1}$, and the process is repeated from step 1.
8) Else, $x_{n*}$ is rejected as a solution and the process is repeated from $x_n$ and $\partial_{U,n}$ at step 1.

Jump length, burn-in length, and random walk durations were parameterized similarly to that for the standard MCMC approach. We used 0.02 as a value for $jmp_{15}$ to allow rapid sampling of the possible solution space. Our code can be downloaded from GitHub at: https://github.com/stukel-lab/N15-LIM.
