# Supplementary figures and images for: A new approach for incorporating 15N isotopic data into linear inverse ecosystem models with Markov Chain Monte Carlo sampling
Source: PLoS One. 2018 Jun 18;13(6):e0199123. doi: 10.1371/journal.pone.0199123 (PMC6005467; doi:10.1371/journal.pone.0199123)

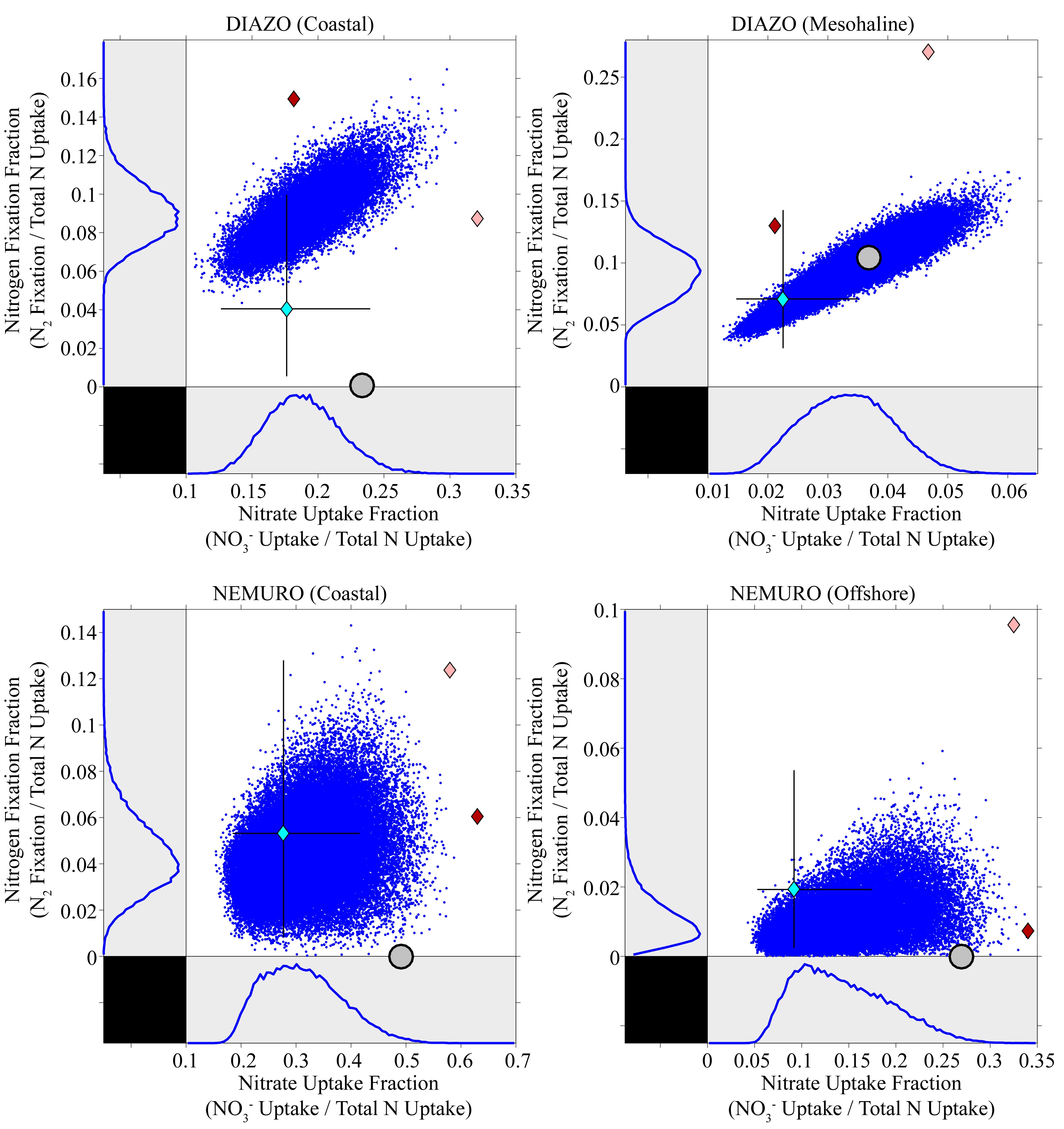

Supplement: S1 Fig — For comparison, the MCMC approach mean value is shown in cyan diamond (with 95% confidence interval) and L2MN and L2MN+15N values are shown in pink and dark red, respectively. (JPG) [file pone.0199123.s002.jpg]

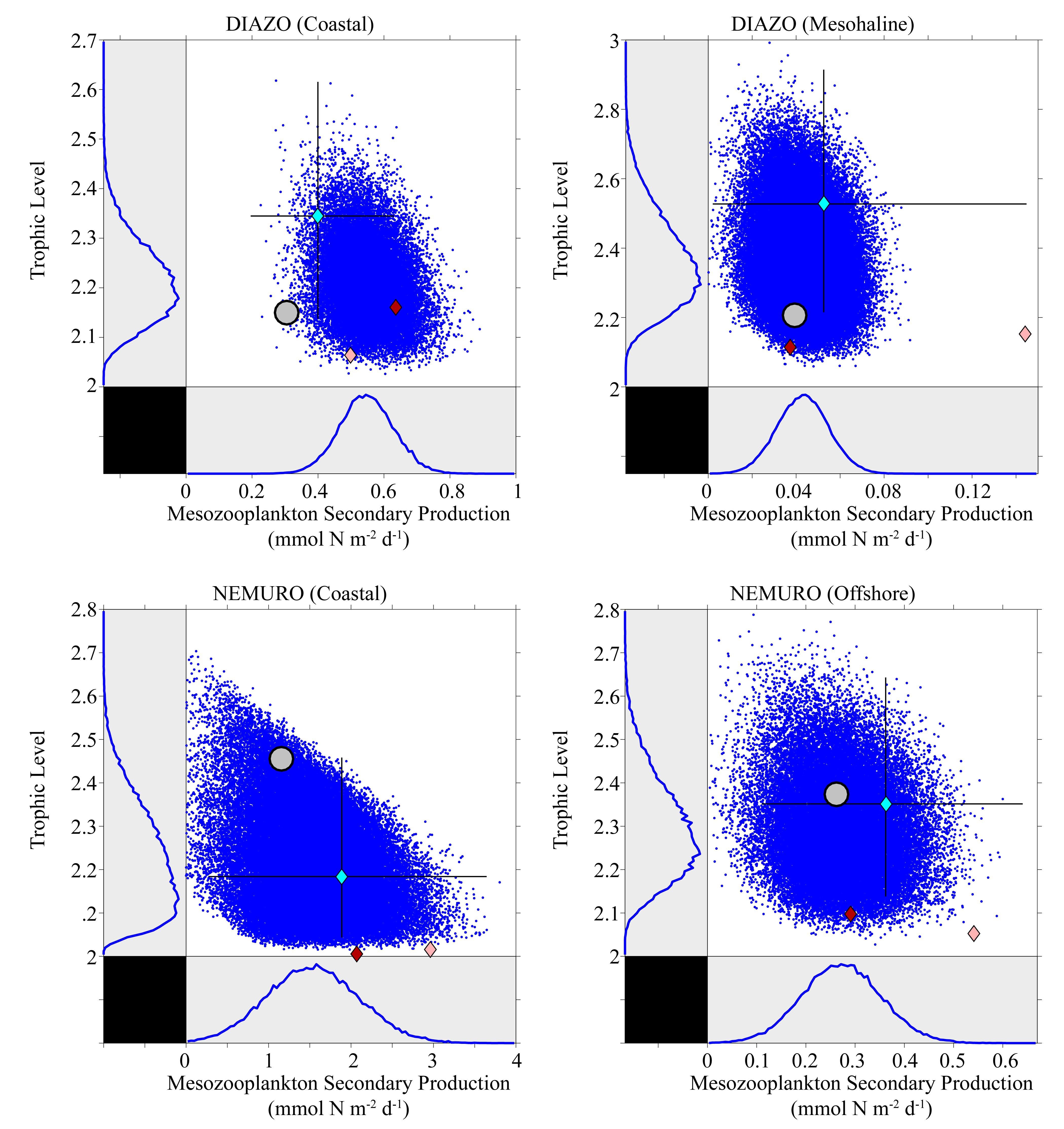

Supplement: S2 Fig — For comparison, the MCMC approach mean value is shown in cyan diamond (with 95% confidence interval) and L2MN and L2MN+15N values are shown in pink and dark red, respectively. (JPG) [file pone.0199123.s003.jpg]
